# Multistage stochastic programming modeling for farmland irrigation management under uncertainty

**DOI:** 10.1371/journal.pone.0233723

**Published:** 2020-06-02

**Authors:** Qi Li, Guiping Hu

**Affiliations:** 1 Department of Mechanical Engineering, Beijing Jiaotong University, Beijing, China; 2 Department of Industrial and Manufacturing Systems Engineering, Iowa State University, Ames, IA, United States of America; Shandong University of Science and Technology, CHINA

## Abstract

Farmland management and irrigation scheduling are vital to a productive agricultural economy. A multistage stochastic programming model is proposed to maximize farmers’ annual profit under uncertainty. The uncertainties considered include crop prices, irrigation water availability, and precipitation. During the first stage, pre-season decisions including seed type and plant density are made, while determinations of when to irrigate and how much water to be used for each irrigation are made in the later stages. The presented case study, based on a farm in Nebraska, U.S.A., showed that a 10% profit increase could be achieved by taking the corn price and irrigation water availability uncertainties into consideration using two-stage stochastic programming. An additional 13% profit increase could be achieved by taking precipitation uncertainty into consideration using multistage stochastic programming. The stochastic model outperforms the deterministic model, especially when there are limited water supplies. These results indicate that multistage stochastic programming is a promising method for farm-scale irrigation management and can increase farm profitability.

## Introduction

As the world population increases and the amount of arable land decreases, it becomes vital to improve the productivity of available farmland. During recent decades, the advent of diesel and electric motors has led to systems that can pump groundwater out of major aquifers and help increase crop productivity. However, concerns have been raised regarding the permanent loss of aquifer capacity, declining surface and groundwater supplies, and increased pumping costs [1, 2]. Thus, irrigation management practices under limited water supplies are critical for sustainable agriculture and food security.

Evapotranspiration (ET) is defined as the water removed from the soil by evaporation from the soil surface and transpiration by plants. ET is driven by atmospheric conditions that exert a drying force on soil or plant surfaces. Hence, the magnitude of daily ET will vary with atmospheric conditions. High solar radiation and air temperatures, low humidity, and high wind increase ET, while cloudy, cool and calm days reduce ET. ET is also affected by crop growth stage, length of growing season, soil fertility, water availability, and the interactions of these factors [3]. When water supplies cannot fully match crop ET requirements, yields are reduced compared to a fully irrigated crop. Under water-limited conditions, yields typically display a positive correlation with the total seasonal ET. On the other hand, applying additional irrigation beyond seasonal ET requirements can lead to leaching and/or water-logging.

Corn is the most widely adopted row crop in the U.S.A. and takes up to one-third of the cropland nationwide. Eighty-seven percent of irrigated corn in the U.S.A. is grown in high or extremely high water stress regions such as the Great Plains and the Central Valley in California. Corn occupies more irrigated acres in these areas than any other crops [4] and receives the most irrigation water among all of the U.S.A. crops [5]. The growth process of corn can be roughly divided into five stages: establishment, vegetative, flowering, grain filling, and ripening. It is worth noting that there is a formal growth stage standard that has several stages divided into vegetative and reproductive stages. Corn is relatively insensitive to water deficits during early vegetative growth and ripening periods because the water demand is relatively low. However, corn is much more sensitive to water stress from flowering through grain-filling stages [6]. Severe water deficits during these periods will cause reduced yield. On the other hand, water-logging should also be avoided, particularly during the flowering and grain-filling stages.

Key factors that affect the irrigation scheduling decisions include soil characteristics, plant features, irrigation methods, and atmospheric factors. Soil characteristics, such as water holding capacity and infiltration capacity, can affect water movement. In addition, some root-restricting layers, such as compaction layer, impermeable layer, or gravel layer, can also restrict root development. Some plant features (phenotypes), such as rooting depth and crop seasonal ET, will affect the drought tolerance (the crop yield response factor to water). For example, rooting depth is related to the ability of extracting water from soil, and crop seasonal ET will affect the water demand of a plant. Selecting an appropriate plant population is as important as choosing a suitable seed type. The main trade-off for plant population is between the increase of seed cost and increase of profit by higher yields. In addition, low plant population is recommended at water-limited sites based on field studies. Irrigation methods determine irrigation application efficiency. Center pivot sprinkler systems can achieve an efficiency of up to 90 percent. However, conventional gated pipe irrigation systems have an application efficiency of only 50 percent, meaning that only half of the water could be utilized by the plant. The rest of the water is lost via drift and droplet evaporation (sprinkler irrigation), runoff, and sometimes deep percolation (leaching). A portion of the lost water could go to aquifer recharge, which is not preferable not only for economic considerations but also for environmental considerations since the unutilized water will carry nitrogen to the aquifer [7].

As a multi-constraint problem, irrigation scheduling is highly affected by the uncertainty from environment, market, and policy. For example, uncertainty in the timing and amount of natural precipitation is the key issue for irrigation scheduling. Moreover, factors such as crop prices and irrigation water availability are also stochastic in nature in semi-arid areas. Farming activities are highly affected by these uncertainties. Thus, decision-making tools for farmland management and irrigation scheduling are particularly necessary.

In summary, the effect of limited water on crop grain yield is significant, and appropriate decisions are needed to optimize farmers’ profits, particularly under stochastic environments. In this study, a multistage stochastic programming model is formulated to maximize annual farm-level net profits by considering uncertainties such as crop prices, precipitation amount, and irrigation water availability. The first stage makes the pre-season decisions, while the later stages determine the irrigation schedule. The main objective of this study is to provide decision support for choosing irrigation and agronomic practices based on the proposed model and to verify the benefit of handling irrigation management under uncertainty via multistage stochastic programming and the significance of considering uncertainty.

## State of the art

In this section, current irrigation practices used in semi-arid areas are introduced. The works in the literature that focus on farmland irrigation management modeling based on mathematical programming are reviewed. The core techniques in stochastic programming, such as scenario generation and measures of information, are also discussed.

### Irrigation practices

Deficit irrigation should be considered in areas where precipitation is low and irrigation water supply is restricted. Deficit irrigation refers to an irrigation strategy in which irrigation is mainly applied during drought-sensitive growth stages of a crop [8]. The understanding of crop yield response functions and the economic impact of reductions in harvest are essential for the correct application of deficit irrigation [9]. Reasons for limited water supplies include but are not limited to, restricted irrigation well capacity, restricted pumping allocations, and limited surface water supplies. Supply, rather than the price of water, is the usual constraint in making irrigation practice decisions [10]. For the majority of farmers, deficit irrigation is used as a strategy to maximize the value of limited water input rather than maximizing the return to land [11].

Classical crop yield response functions are employed to reflect the impacts of various deficit levels on crop yield [12]:

$$\begin{array}{c} \frac{Y_{a}}{Y_{m}}=\prod_{j=1}^{J}\left[1-k_{j}\left(1-\frac{ETa_{j}}{ETc_{j}}\right)\right] \end{array}$$ (1)

where *Y*_*a*_ is the actual crop yield, *Y*_*m*_ is the maximum crop yield under full irrigation, *k*_*j*_ is the crop yield response factor to water that is a function of the crop type and the stage of growth, *J* is the total number of crop growth stages, *ETa*_*j*_ is the actual crop evapotranspiration at stage *j*, and *ETc*_*j*_ is the crop evapotranspiration without water stress at stage *j*. If a single or integrated crop growth stage is considered, Eq (1) could be reduced to a version without multiplication and index *j*.

Irrigation scheduling is of vital importance under conditions of marginal rainfall and limited irrigation water supplies. For deficit irrigation of corn, it is suggested that water could be saved to the flowering and grain-filling stages, since corn is much more sensitive to water stress from flowering through the grain-filling stage. However, irrigation schedules are sometimes relatively simple and crude in practice, with the same amount of irrigation water applied at equal time intervals. Precision management of irrigation frequency and quantity is needed, especially in semi-arid areas.

Establishing advanced irrigation systems is an important approach for farmland water management under deficit irrigation. Pressure systems and gravity systems are two main categories of irrigation systems that are based on energy and pressure requirements [13]. Pressurized irrigation systems make up roughly 58-65% of irrigation systems used in the U.S.A. [14]. Pressurized irrigation systems include center pivot, linear move, hand move, solid set, drip irrigation, and low-flow micro sprinklers. In the U.S.A., the 2014 Farm Bill distributed $1.2 billion in funding toward implementing irrigation systems between 2009 and 2014, and nearly half of that amount went toward implementing sprinkler and micro-irrigation systems [14]. Advanced process control strategies, such as model-based control, are other useful tools for irrigation management [15]. Other technologies, such as satellite data, sensor networks, data analytics, and unmanned aircraft systems, may also increase irrigation efficiency and increase crop yield [16]. These technologies have become the basis for precision agriculture and farmland risk management.

### Irrigation management models based on mathematical programming

Mathematical programming, and stochastic programming in particular, has been widely applied to (large-scale) irrigation management [17]. Stochastic programming is a mathematical programming method where some of the parameters in the objective function and/or constraints are uncertain. Li et al. [18] developed an inexact two-stage stochastic programming model for river basin water resources planning under uncertainty. Jin and Huang [19] extended this work to a robust inexact fuzzy set linear programming model for irrigation water systems. Li et al. [20] presented a fuzzy two-stage stochastic programming approach for water allocation problems at the county level, considering economic benefits and policy penalties. The model applied the concepts of interval-parameter and fuzzy programming techniques when the parameter distribution was not known. Li et al [21] applied a hybrid methodology of conditional value-at-risk measure, a general two-stage stochastic programming framework, and interval-parameter programming to solve water resources allocation problems. Robert et al. [22] used a stochastic dynamic programming model for regional-scale groundwater irrigation management considering farmers’ adaptation decisions. Yang et al. [23] applied interval-parameter programming to model the irrigation water allocation problem on a regional scale, and the priority order of crop selection was given. Artificial intelligence and meta-heuristic methods are also useful for irrigation management. Zhang et al. [24] applied genetic algorithms and non-linear optimization to corn irrigation considering stages of crop growth, the grain market price, irrigation water price, minimum yield, and irrigation cost etc. Kontos and Katsifarakis [25] employed genetic algorithms for irrigation and drinking water management for coastal aquifer. Jimenez et al. [26] employed Long Short-Term Memory Neural Network for precision irrigation considering different soil types. In summary, these studies highlighted multiple constraints and uncertainty or risk analysis in irrigation management. These studies focus on certain source of uncertainty from economic, resources, and policy aspects. However, farmland irrigation scheduling under multiple uncertainties such as crop prices, precipitation amount, and irrigation water availability have not been studied extensively. Furthermore, most of these studies focused on irrigation water allocation problems on the regional scale rather than the farm scale.

Farm scale land management and irrigation scheduling have been the subject of research studies as well. Ganji et al. [27] proposed a constraint state formulation for a weekly deficit irrigation strategy under stochastic conditions. The model was based on the first and second moment analysis of the stochastic soil moisture state variable and considered the crop water demand uncertainty. Brown et al. [28] used simulated annealing for on-farm irrigation scheduling considering seasonal water limits. The model described general irrigation strategies for a multicrop irrigation scheduling problem, and time-series simulation of climate stochastic characteristics was employed to deal with uncertainty. They argued that a 10% profit increase could be achieved if the primary constraint on water availability was the seasonal water limitation rather than water price, which restricted the maximum number of irrigation events in a season. Ridier et al. [29] applied a dynamic stochastic programming model for crop rotation at the farm level, in which market risk was considered as an inter-year risk while production risk was an intra-year risk. Li et al. [30] presented a farm-level precision land management framework based on integer programming. They considered corn market prices and irrigation water prices by using sensitivity analysis. However, to the best of the authors’ knowledge, few applications of farm-scale irrigation management based on multistage stochastic programming have been reported. Therefore, the objective of this study is to investigate the feasibility and advantage of modeling farm-scale land management and irrigation scheduling via stochastic programming. In addition, most studies have used two-stage stochastic programming frameworks, and the advantage of using multistage stochastic programming should also be discussed.

## Motivating data

In many advanced agricultural applications, farm monitoring enables the use of real-time observations in the decision-making process. However, many farms in semi-arid areas still use average crop prices and the average ET amount to make pre-season decisions and irrigation schedules all at once at the beginning of the year.

In this study, a multistage stochastic programming model is formulated considering uncertainties such as crop prices, precipitation amount, and irrigation water availability. These uncertainties are represented by scenario trees as realizations of probability distributions or stochastic processes. The objective is to maximize a farmer’s annual net profit by finding the optimal decisions for the pre-season decisions and irrigation schedules. In this study, there are nine time periods (*t* = 0, 1, …, 8;) considered in the model. The time period 0 (*t* = 0) is at the beginning of the year, and the time period 1 (*t* = 1) is at the beginning of the corn flowering stage. The time period 1 to 8 (*t* = 1, 2, …, 8;) corresponds to the eight weeks for the flowering and grain-filling stages of corn. The crop price and seasonal irrigation water availability information are assumed to be released at the beginning of time period 1 (*t* = 1). Precipitation information of these eight weeks is available at the end of each time period.

**Fig 1:**
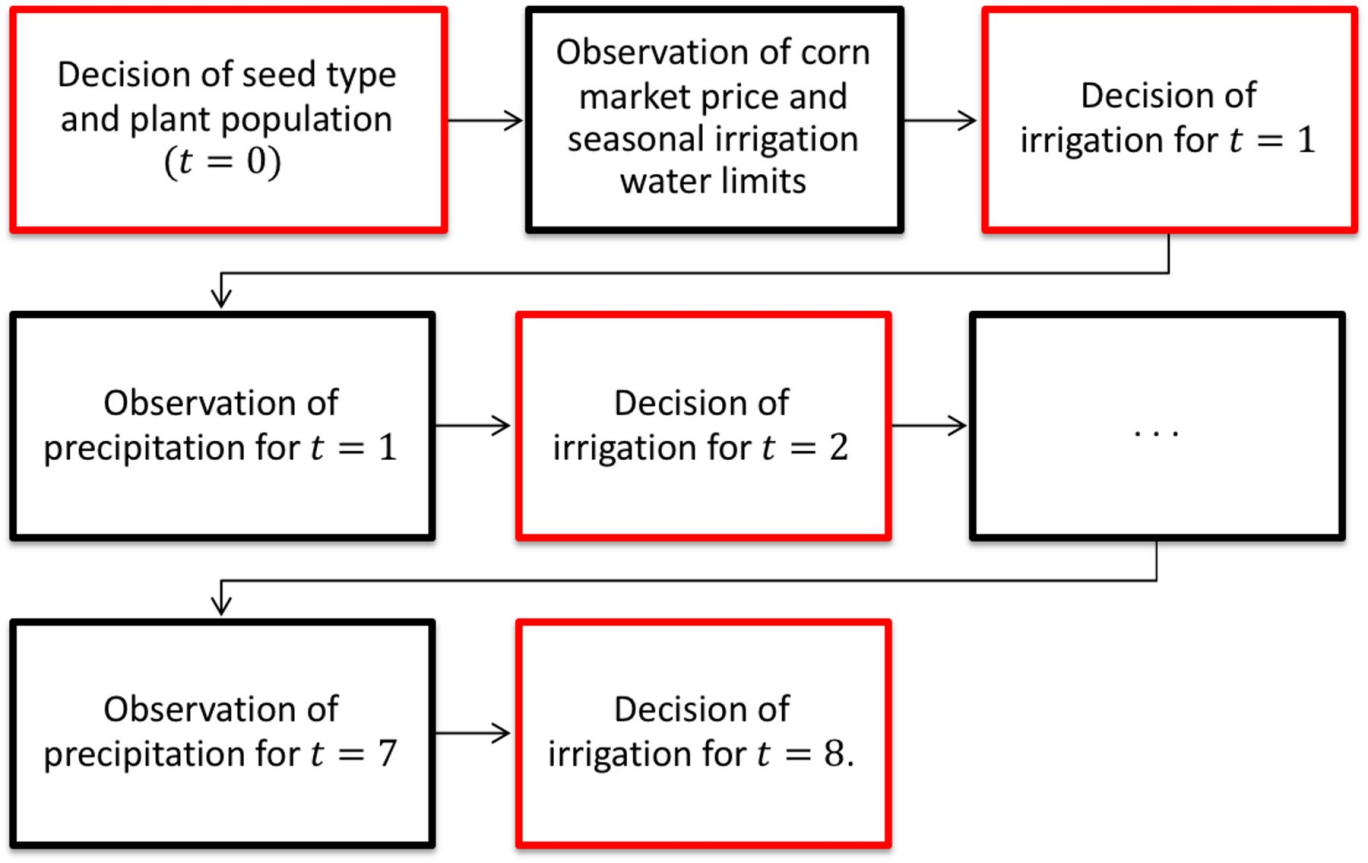
The detailed decision process for multistage stochastic programming.

The decision maker makes a sequence of decisions at each time period in order to maximize profit. In this problem, the decision maker makes the pre-season decisions including the corn seed type selection and the plant population selection at the first stage (*t* = 0). At the beginning of the second stage (*t* = 1), realizations of corn price and seasonal irrigation water availability become available, and the second-stage decisions of how much irrigation water should be applied in week one (*t* = 1) are made. At the beginning of the 2^nd^ to the 8^th^ time period, similar irrigation scheduling decisions are made based on sequentially released information. The detailed decision process and information release process are shown in Fig 1.

Since corn is more sensitive to water during the flowering and grain-filling stages, it is assumed that irrigation will only take place in the flowering and grain-filling stages. An integrated crop yield response factor for the flowering and grain-filling stages is used. The decision maker can decide to apply less than the normal amount of water for each (weekly) irrigation during the flowering and grain-filling stages to maximize the farmer’s annual net profit. However, it is worth noting that it would be important to irrigate in the early season when there is less early-season precipitation [31].

## Methods

Techniques for stochastic programming are first introduced, and then the multistage stochastic programming model is presented to address decision making under uncertainty. The model was formulated in The General Algebraic Modeling System (GAMS) 23.4.3 from GAMS company and solved using CPLEX Optimizer 12.1.0 from IBM company. GAMS is a modeling software that formulate optimization problems in a notation similar to their algebraic notation [32]. IBM CPLEX Optimizer is free for academia, it provides flexible, high-performance mathematical programming solvers for linear programming, mixed integer programming. Interested party can obtain these tools via the links in the supporting materials.

### Techniques for stochastic programming

In stochastic programming frameworks, the decision maker makes certain decisions at the first stage. The outcomes of these decisions will be affected by random events, with the later stage decisions made to adjust for these effects. In other words, stochastic programming provides first-stage decisions and a collection of subsequent (recourse) decisions based on each random outcome. It reflects the dynamic system, especially for sequential decision-making problems [33].

Computational methods for solving stochastic optimization problems require a discretization (in the form of scenario trees) of the underlying probability distribution or process of the uncertain parameters. The sample average approximation (SAA) method [34] based on probability distributions and the moment matching method [35] of historical data are often employed for scenario generation.

Multistage stochastic programming is more flexible than two-stage stochastic programming. Thus, measures of information are needed to discuss the value of stochastic programming and information. In two-stage stochastic programming, standard approaches based on different indicators have been detailed in textbooks and are widely found in the literature [36].

In the content of stochastic programming, a “decision” refers to a set of actions (e.g., irrigation scheduling) and a “solution value” (or “solution” in short) refers to “the value of the objective function” (e.g., profit). The “expected value problem” (*EV*) is a deterministic model that replaces all random variables by their expected values. Thus, “*EV* solution” refers to a number while “*EV* decision” refers to a set of decisions that could also be applied in stochastic environments. When the *EV* decision is applied in different scenarios, it will return with a set of solution values. The average of these solution values is referred as “the expectation of expected value problem solution” (*EEV*). *EEV* measures the performance of deterministic decisions in stochastic environments. Instead of using the same decision for all scenarios, it hypothetically finds its own optimal decisions for each scenario and obtains its solution values. The weighted average of these solution values is referred to as the wait-and-see (*WS*) solution value. Correspondingly, the solution of the stochastic model, also known as the here-and-now solution, denotes the optimal solution value for the recourse problem (*RP*). To avoid confusion, unless otherwise stated, *EV*, *EEV*, *WS*, and *RP* all refer to the value of the objective function. For maximization problems, the following inequalities are satisfied:

$$\begin{array}{c} EEV\leq RP\leq WS \end{array}$$ (2)

There are mainly two indicators for measuring information in two-stage stochastic programming, the expected value of perfect information (*EVPI*) and the value of the stochastic solution (*VSS*). In this context, *EVPI* = *WS* − *RP* compares here-and-now and wait-and-see approaches; a large *EVPI* means a large additional profit with sufficient information. *VSS* = *RP* − *EEV* compares the here-and-now and expected values approaches. A large *VSS* means that the stochastic programming approach is able to take advantage of taking uncertainty into account in decision making.

The *WS* is still valid in multistage stochastic programming frameworks, in which the decision makers assume to know the realizations of all the random variables at the first stage. However, the *EEV* for multistage stochastic programming is sometimes misleading [37]. Thus, the value of multistage stochastic programming (*VMS*), which is the difference between the optimal objective values of the two-stage $\left(v_{r}^{TS}\right)$ and multistage formulations $\left(v_{f}^{MS}\right)$, is adopted in this study:

$$\begin{array}{c} VMS=v_{f}^{MS}-v_{r}^{TS} \end{array}$$ (3)

To avoid confusion, let *EEV*^*TS*^ be the expectation of the expected value problem solution in two-stage stochastic programming. The relative values of two-stage stochastic programming (*RVSS*) and multistage stochastic programming (*RVMS*) are defined as follows [38]:

$$\begin{array}{c} RVSS=\left(v_{f}^{TS}-EEV^{TS}\right)/EEV^{TS} \end{array}$$ (4)

$$\begin{array}{c} RVMS=\left(v_{f}^{MS}-v_{r}^{TS}\right)/v_{r}^{TS} \end{array}$$ (5)

### Deterministic model

A mixed integer linear programming model is first formulated, and all the system parameters are assumed to be known with certainty in the deterministic model. The objective is to maximize a farmer’s annual net profit, which is defined as total revenue subtracted by total system costs. The binary decision variable *x*_*i*_ represents which pre-season management option *i* is used, and *x*_*i*_ = 1 means the option *i* is used. The positive decision variable *y*_*t*_ represents the net irrigation (i.e., the irrigation water that is used by the crop) during time period *t*. The binary variable *z*_*t*_, which is dependent on *y*_*t*_, represents whether irrigation is performed during time period *t*. The objective function is defined as follows:

$$\begin{array}{c} max\;GA\sum_{l=1}^{L}Y_{l}^{c}-AC^{wt}\left(\sum_{t=1}^{T}y_{t}/\gamma +W^{p}\right)-AC^{f}\sum_{t=1}^{T}z_{t}-A\sum_{i=1}^{I}x_{i}\left(C_{i}^{s}+C_{i}^{m}\right)-C^{o} \end{array}$$ (6)

where *G* is the unit market corn price, *A* is the total area of the farmland, and $Y_{l}^{c}$ is the actual yield under deficit level *l*. Thus, $GA\sum_{l=1}^{L}Y_{l}^{c}$ is the annual revenue. A variety of system costs have been considered in the model including labor costs, irrigation costs, machinery costs, seed costs, chemicals costs, cash overhead, and non-cash overhead. Irrigation costs consist of two parts. The first part is the water purchasing costs, including the pre-season irrigation, represented by $AC^{wt}\left(\sum_{t=1}^{T}y_{t}/\gamma +W^{p}\right)$, where *C*^*wt*^ is the unit cost for purchased water, *γ* is the irrigation application efficiency, and *W*^*p*^ is the pre-irrigation water amount per acre. This part of cost is changeable based on the irrigation amount. The second part, represented by $AC^{f}\sum_{t=1}^{T}z_{t}$, is the fixed portion of irrigation costs per time. *C*^*f*^ is the unit fixed cost for irrigation, which includes the costs of labor and equipment for each irrigation process. To have a concise expression and focus on the impacts of irrigation management, several farm operating costs, including labor costs (not including irrigation labor cost), machinery costs, and chemicals costs, are lumped together and called “other farm operating costs”. The “other farm operating costs” and the seed costs together are represented by $A\sum_{i=1}^{I}x_{i}\left(C_{i}^{s}+C_{i}^{m}\right)$, where $C_{i}^{s}$ is the unit cost for the purchase of seed under pre-season management option *i*, and $C_{i}^{m}$ is the unit “other farm operating costs” under pre-season management option *i*. Cash overhead consists of various cash expenses that are assigned to the whole farm, such as insurance, office expenses, machinery maintenance, property tax, and field supervisors’ salary. Non-cash overhead includes capital recovery cost (annual depreciation and interest costs) for equipment and other farm investments. Cash and non-cash overhead costs are represented by *C*^*o*^.

The following two constraints are the soil moisture continuity equations for the time periods:

$$\begin{array}{c} M_{t}+y_{t}+R_{t}-ET_{t}^{a}-L_{t}^{W}=M_{t+1}\;for\;t=1,2,\cdots 7 \end{array}$$ (7)

$$\begin{array}{c} M_{t}+y_{t}+R_{t}-ET_{t}^{a}-L_{t}^{W}\geq 0\;for\;t=8 \end{array}$$ (8)

where *M*_*t*_ represents the water available in the soil at the beginning of time period *t*, *R*_*t*_ represents the total precipitation during time period *t*, $ET_{t}^{a}$ represents the actual evapotranspiration during time period *t*, and $L_{t}^{W}$ is the leaching water amount during time period *t*. The positive decision variable *y*_*t*_ represents the net irrigation during time period *t*. For each time period, irrigation and precipitation will replenish soil moisture, while *ET* and leaching will consume water. Irrigation and precipitation plus current soil moisture should be less than the soil water holding capacity; otherwise, the extra water that will leach is wasted. This constraint is reflected by the following:

$$\begin{array}{c} M_{t}+y_{t}+R_{t}-L_{t}^{W}\leq H\;\forall t \end{array}$$ (9)

where *H* represents the soil water holding capacity.

The deficit level is defined as the ratio between actual evapotranspiration and the evapotranspiration without any water stress. The definition of deficit level is reflected by the following:

$$\begin{array}{c} \sum_{t=1}^{T}ET_{t}^{a}/ET_{t}^{m}=\sum_{l=1}^{L}d_{l}D_{l} \end{array}$$ (10)

where $ET_{t}^{m}$ is the crop stage evapotranspiration without any water stress during time period *t*, and $ET_{t}^{a}$ is the actual crop stage evapotranspiration during time period *t*. *d*_*l*_, as binary variables, represent whether deficit level *l* is applied, and *D*_*l*_ is the percentage of the maximum crop stage evapotranspiration achieved in deficit level *l*. To have a smooth change among deficit levels, 101 equidistant deficit levels from 0% to 100% are used. For example, if *d*_100_ equals 1, the right-hand side of the constraint in Eq (10) will be equal to 0.99.

The crop yield response functions for water usage based on Eq (1) are reflected by the following two equations:

$$\begin{array}{c} Y_{l}^{c}-\sum_{i=1}^{I}x_{i}Y_{i}^{m}\left(1-K_{i}\left(1-D_{l}\right)\right)\leq \left(1-d_{l}\right)M^{b}\;\forall l \end{array}$$ (11)

$$\begin{array}{c} Y_{l}^{c}-\sum_{i=1}^{I}x_{i}Y_{i}^{m}\left(1-K_{i}\left(1-D_{l}\right)\right)\geq \left(d_{l}-1\right)M^{b}\;\forall l \end{array}$$ (12)

where $Y_{l}^{c}$ represents the actual crop yields under deficit level *l*, and $Y_{i}^{m}$ represents the maximum unit crop yield when management option *i* is used. *K*_*i*_ represents the crop yield response factor to water during flowering and grain-filling stages under pre-season management option *i*. *M*^*b*^ is a sufficiently large number used in the “big-M method” [39]. For computation considerations, the *M*^*b*^ should be as small as possible, and it is set to be equal to $\text{max}Y_{l}^{c}$. Only one deficit level should be selected, and this requirement is presented in the following constraint by using binary variable *d*_*l*_:

$$\begin{array}{c} \sum_{l=1}^{L}d_{l}=1 \end{array}$$ (13)

Constraints in Eqs (11) to (13) together ensure “only one out of *L* constraints must hold”. For example, when *d*_*l*_ = 1, the right-hand side of the constraints in Eqs (11) and (12) will equal zero, and these two constraints will yield $Y_{l}^{c}=\sum_{i=1}^{I}x_{i}Y_{i}^{m}\left(1-K_{i}\left(1-D_{l}\right)\right)$; when *d*_*l*_ = 0, Eqs (11) and (12) become relaxation constraints.

The following constraint ensures only the chosen deficit level will lead to reasonable (positive) actual crop yields:

$$\begin{array}{c} d_{l}M^{b}\geq Y_{l}^{c}\;\forall l \end{array}$$ (14)

As a vulnerable and valuable resource, the amount of irrigation water is often limited in key growing stages. This irrigation water limitation is reflected in the following constraint:

$$\begin{array}{c} A\sum_{t=1}^{T}y_{t}/\gamma \leq W^{l} \end{array}$$ (15)

where *W*^*l*^ is the total irrigation water limitations during flowering and grain-filling stages.

For the consideration of food safety and market stability, the government will sometimes encourage farmers to produce at least a certain amount of crops. A similar total yield constraint is needed when there is a commercial contract including a yield mandate. These situations are indicated in the following constraint:

$$\begin{array}{c} A\sum_{l=1}^{L}Y_{l}^{c}\geq Y \end{array}$$ (16)

where *Y* is the minimum yield requirements for the farmland.

The total frequencies of irrigation are needed to calculate the cumulative fixed costs of labor and equipment for irrigation. These costs occur only if the irrigation actually takes place (irrigation water amount is above zero), as reflected in the following constraint:

$$\begin{array}{c} M^{b}z_{t}\geq y_{t}\;\forall t \end{array}$$ (17)

This is another application of the “big-M method”. Since *M*^*b*^ is sufficiently large, *z*_*t*_ = 1 will lead to no constraint for *y*_*t*_, and *z*_*t*_ = 0 will lead to *y*_*t*_ = 0.

As shown in the following constraint, only one seed type and plant population should be selected:.

$$\begin{array}{c} \sum_{i=1}^{I}x_{i}=1 \end{array}$$ (18)

The following constraint makes a conservative assumption that there is no water in the soil at the beginning of the first time period, though the soil is likely not quite that dry for most years:

$$\begin{array}{c} M_{t}=0\;for\;t=1 \end{array}$$ (19)

The domain of variables is controlled by the following constraint:

$$\begin{array}{c} i,t,l\in N,d_{l}\in \left\{0,1\right\},x_{i},\;y_{t},Y_{l}^{c},M_{t},ET_{t}^{a},L_{t}^{W}\geq 0\;\forall i,\forall t,\forall l \end{array}$$ (20)

### Multistage stochastic programming model

In this study, precipitation amount, irrigation water availability, and corn prices are selected as the stochastic parameters to be investigated. Scenario trees are used as an approximation of probability distributions or stochastic processes. Subscript *w* is used to represent the index of the scenario with corresponding probability *P*_*w*_, and this subscript is also incorporated into the decision variables and parameters. The multistage stochastic programming model is formulated as follows:

$$\begin{array}{c} max\;-A\sum_{i=1}^{I}x_{i}\left(C_{i}^{s}+C_{i}^{m}\right)-C^{o}+\sum_{w=1}^{W}P_{w}\left\{G_{w}A\sum_{l=1}^{L}Y_{lw}^{c}-AC^{wt}\left(\sum_{t=1}^{T}y_{tw}/\gamma +W^{p}\right)-AC^{f}\sum_{t=1}^{T}z_{tw}\right\} \end{array}$$ (21)

s.t.

$$\begin{array}{c} M_{tw}+y_{tw}+R_{tw}-ET_{tw}^{a}-L_{tw}^{W}=M_{t+1,w}\;\forall w,t\in \left\{1,2,\ldots 7\right\} \end{array}$$ (22)

$$\begin{array}{c} M_{tw}+y_{tw}+R_{tw}-ET_{tw}^{a}-L_{tw}^{W}\geq 0\;\forall w,t=8 \end{array}$$ (23)

$$\begin{array}{c} M_{tw}+y_{tw}+R_{tw}-L_{tw}^{W}\leq H\;\forall t,\forall w \end{array}$$ (24)

$$\begin{array}{c} \sum_{t=1}^{T}ET_{tw}^{a}/ET_{t}^{m}=\sum_{l=1}^{L}d_{lw}D_{l}\;\forall w \end{array}$$ (25)

$$\begin{array}{c} Y_{lw}^{c}-\sum_{i=1}^{I}x_{i}Y_{i}^{m}\left(1-K_{i}\left(1-D_{l}\right)\right)\leq \left(1-d_{lw}\right)M^{b}\;\forall l,\forall w \end{array}$$ (26)

$$\begin{array}{c} Y_{lw}^{c}-\sum_{i=1}^{I}x_{i}Y_{i}^{m}\left(1-K_{i}\left(1-D_{l}\right)\right)\geq \left(d_{lw}-1\right)M^{b}\;\forall l,\forall w \end{array}$$ (27)

$$\begin{array}{c} \sum_{l=1}^{L}d_{lw}=1\;\forall w \end{array}$$ (28)

$$\begin{array}{c} d_{lw}M^{b}\geq Y_{lw}^{c}\;\forall l,\forall w \end{array}$$ (29)

$$\begin{array}{c} A\sum_{t=1}^{T}y_{tw}/\gamma \leq W_{w}^{l}\;\forall w \end{array}$$ (30)

$$\begin{array}{c} A\sum_{l=1}^{L}Y_{lw}^{c}\geq Y\;\forall w \end{array}$$ (31)

$$\begin{array}{c} M^{b}z_{tw}\geq y_{tw}\;\forall t,\forall w \end{array}$$ (32)

$$\begin{array}{c} \sum_{i=1}^{I}x_{i}=1 \end{array}$$ (33)

$$\begin{array}{c} M_{tw}=0\;for\;t=1\;\forall w \end{array}$$ (34)

$$\begin{array}{c} i,t,l,w\in N,d_{lw}\in \left\{0,1\right\},x_{i},y_{tw},Y_{lw}^{c},M_{tw},ET_{tw}^{a},L_{tw}^{W}\geq 0\;\forall i,\forall t,\forall l,\forall w \end{array}$$ (35)

$$\begin{array}{c} y_{tw}=y_{tw^{\prime }}\;\forall t,\forall w,w^{\prime }\;for\;which\;\xi_{\left[t\right]}^{w}=\xi_{\left[t\right]}^{w^{\prime }} \end{array}$$ (36)

The first-stage decisions are made before uncertainties are realized. After uncertainties are progressively realized, the decisions of later stages are made. In this model, *x*_*i*_ is the first-stage decision variable, and *y*_*tw*_ is the later-stage decision variable. The constraint in Eq (33) is the first-stage constraint, which remains the same in all scenarios. The rest of the constraints change based on stochastic scenarios. We use notation *ξ*_*t*_(*t* ∈ {1, …, *T* − 1}) to denote a random vector and its particular realization at each time period. The decision at each period (*t* ∈ {1, …, *T*}) depends on the realizations of *ξ*_*t*_ up to time *t*. Generally, at stage *t* ∈ {1, …, *T*}, scenarios that have the same history *ξ*_[*t*]_ cannot be distinguished, so we need to enforce the “nonanticipativity constraints” by adding Eq (36).

**Fig 2:**
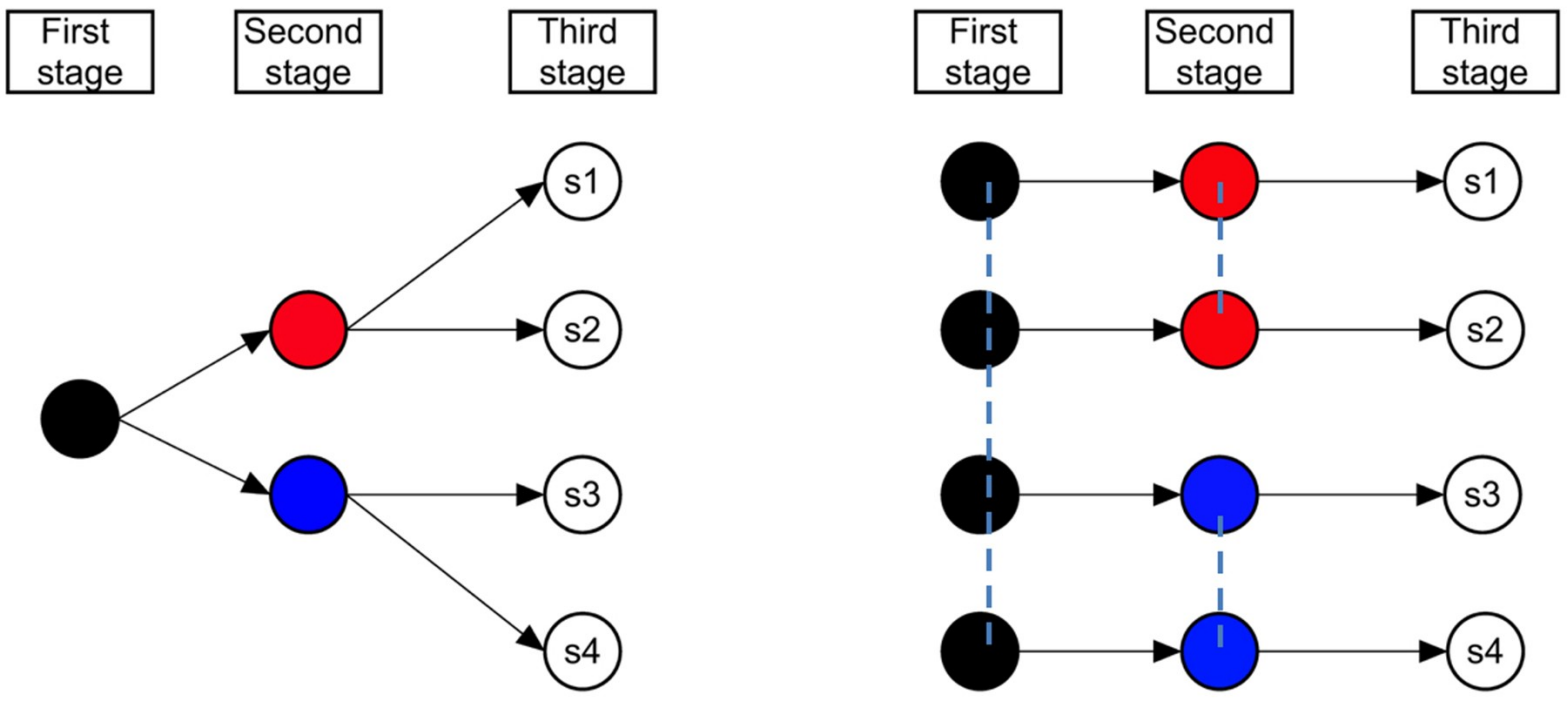
Decision tree example with two periods, three stages, and four scenarios.

Fig 2 shows a decision tree example with 2 periods, 3 stages, and 4 scenarios. The arrow represents the period, the branch represents the scenario, and stages are labeled. The left part of Fig 2 is a tree shape, while the right part is a fan shape. These two types of forms are equivalent. The “nonanticipativity constraints” are represented by the dashed lines in Fig 2, which ensures scenarios with the same history should have the same decisions at that stage. In this example, all four scenarios have the same first-stage decisions, scenario one and scenario two have the same second-stage decisions while scenario three and scenario four have the same second-stage decisions.

## Results and discussion

The authors apply the irrigation management frameworks for a case study on a farm in Cherry County, Nebraska, U.S.A., to illustrate and validate the optimization model. Although the High Plains Aquifer under Nebraska is well-managed, Nebraska is the fourth largest user of groundwater in the nation, and its prominence in irrigation agriculture has expanded greatly over the past two decades. Half of the harvested row crop production in Nebraska is irrigated (approximately 3.2 × 10^8^ *m*^2^), where corn occupied approximately 70 percent of the irrigated acreage in 2008 [40]. Cherry County is located at the north part of Nebraska, precipitation is insufficient and deficit irrigation is commonly used in that area. In addition, risk analysis listed Cherry County as one of the most vulnerable areas in Nebraska [41]. Consequently, improving farmland management and irrigation scheduling has significant impacts on water resources utilization and farm profitability, at least in semi-arid areas.

### Data sources

Since all the data are collected from public sources and the farm supply no data to this study, no permission are needed to conduct this case study. The methods of data collection are summarized in this section and related data sets are provided in the supporting materials.

Conservative irrigation management typically assumes an effective root zone of 91 *cm* (three feet) for field corn. Soil information collected by the Web of Soil Survey is used to define integrated soil types (fine sand, loamy sand, sandy loam, fine sandy loam, loam, clay loam, and clay) and their water holding capacities [42]. A farm of 6.0 × 10^5^ *m*^2^ (150 acres) in Cherry County, Nebraska, U.S.A, is selected (101° 12′ W, 42° 53′ N). Approximately 95% of the soil is loamy sand and 5% of the soil is sandy loam, and both of them are coarse soil. The soil water holding capacity is assumed to be 8 *cm* water per *m* soil (1.1 inch per foot) for the whole land [42]. The irrigation water is supplied by center pivot sprinkler systems with a capacity of 0.05 *m*^3^/*s* (800 gallons per minute). The application efficiencies for center pivots outfitted with low pressure drop nozzles are typically rated at 85% [43], meaning that 15% of the water is lost via drift, droplet evaporation, runoff, and sometimes deep percolation (leaching).

The root zone should be wetted at sowing in order to obtain a good germination rate and rapid root development. Thus, pre-irrigation in the spring is needed to refill the soil profile, particularly when there is limited winter precipitation. Since corn does not consume much water in the vegetative stage and does not need much irrigation, this study focuses on the irrigation for flowering and grain-filling stages (approximately eight weeks). The range of average ET for these period is 5 − 8 *mm* per day [40].

The price of irrigation water is volatile and varies significantly by locations, water usages, and water types (groundwater or surface water). In this study, it is assumed that farmers use groundwater at an average abstraction cost of 0.12 $/*m*^3^ (12 $/acre-inch). Other farm operating costs and fixed irrigation costs are adopted from the Nebraska Water Optimizer Single-Field Version (referred to as the NWO model) [44]. Nitrogen fertilizer and pesticide are set to their practical level based on the NWO model for scenarios. Seed features such as drought tolerance and target yields are based on commercialized crop hybrids. For the mix of seed information with plant population, the maximum yield level considered in the model is approximately 1.08 − 1.42*kg*/*m*^2^ (160-210 bushels per acre).

The corn prices received by U.S.A. corn producers from 2000 to 2015 were collected based on the National Agricultural Statistical Service of the U.S.A. Department of Agriculture [45]. The baseline for corn price in the deterministic case is set at 0.14 $/*kg* (3.6 dollars per bushel). Historical precipitation information of Cherry County was obtained from the National Oceanic and Atmospheric Administration’s National Centers for Environmental Information [46]. Detailed discussions on the distribution of corn price, precipitation amount, and total water limits are given in the following scenario generation section. All cost data have been adjusted for inflation to 2015 U.S.A. dollars.

### Scenario generation

Finding the appropriate distribution is critical for scenario generation. Since a single year (the year 2015) problem is considered in this case study, a meaningful corn price should be the average price received by farmers after corn is harvested and ready to sell. The market year of corn sales starts in September, and a six-month sales season is considered. In other words, the distribution of the average corn price from September to the following February is needed. This distribution is assumed to be conditional on the corn price before the sowing season, which is April in Nebraska. The Shapiro-Wilk normality test [47] of the historical corn price data yields a *P*-value of 0.84, meaning that it is reasonable to assume that these conditional data follow a normal distribution. The maximum likelihood method [48] is used to obtain parameter estimations. In summary, the average corn price follows a normal distribution, with the mean equal to the corn price in April minus 0.15 dollars per bushel, and the standard deviation is 0.34.

Precipitation is one of the most important weather variables. The method for precipitation prediction is fairly well established, and reliable simulation techniques are available [49]. In this study, a two-step process is adopted for precipitation generation: the daily precipitation occurrence (i.e., wet or dry day) is modeled with a first-order two-state Markov chain. Once it rains, the precipitation amount is assumed to follow gamma distributions [50, 51]. It is assumed that daily precipitation for each week follows its unique gamma distribution, and the simulation results are then integrated with the daily precipitation occurrence on a weekly basis. Table 1 summarizes the parameter estimations of daily precipitation by week and the average of the integrated weekly total precipitation.

**Table 1:** Parameter estimations of daily precipitation and the integrated total precipitation by week (inch).

Week	Mean	Median	Variance	Integrated weekly rainfall average
1	0.44	0.33	0.15	0.73
2	0.46	0.34	0.19	0.69
3	0.47	0.35	0.17	0.65
4	0.4	0.28	0.16	0.52
5	0.46	0.34	0.18	0.70
6	0.44	0.34	0.15	0.65
7	0.41	0.30	0.15	0.56
8	0.34	0.26	0.08	0.38

Based on a center pivot sprinkler system, the theoretical upper bound for eight weeks of total water availability is 2.4 × 10^5^ *m*^2^ (2355 acre-inches in total or 15.7 inches per acre). However, high application rates of water to coarse textured soils can destroy surface soil structure and increase runoff. Thus, the practical upper bound for total water available is set to be 2.3 × 10^5^ *m*^2^ (2240 acre-inches in total or 14.9 inches per acre) [40]. Political reason such as seasonal water allocation is one of the most important sources for water availability. In some locations, the farmers have been given a limited amount of water that can be used over the season. For example, some districts in Nebraska have water allocations of 12 inches per year. On the other hand, system down time due to maintenance, system failure, insufficient groundwater, and electrical load control should also be taken into consideration. For example, Nebraska Public Power Districts can be authorized to interrupt power for up to six 12-hour periods during a week [42]. In this study, a relatively general lower bound is set to be 1.7 × 10^5^ *m*^2^ (1649 acre-inches in total or 11.0 inches per acre), or 70% of the theoretical upper bound. Since there is an insufficient amount of data to fit a distribution of total water limits, a uniform distribution with a range from 1.7 × 10^5^ *m*^2^ to 2.3 × 10^5^ *m*^2^ (approximately 11-15 inches per acre) is used. However, it is worth noting that if more information is available for seasonal water limitations, the distribution of this stochastic parameter should be revised.

Since the distributions of random variables are available, a common approach to generate the scenario to a manageable size is the SAA method based on Monte Carlo simulation. It is assumed that these three random variables are independent. Scenario tree construction and reduction was based on the method by Heitsch [52], and the size of the scenario is set to be 200 given the computational capacity. The numerical results, interpretations, and stability test are presented in the following results analysis sections.

### Results based on analysis of the deterministic model

The deterministic model yields a total profit of $27,494, which will be used as the objective value of the *EV* problem. Seed with the highest yield and the highest plant population is selected by the model in the deterministic case. This is because under the average total water limits and precipitation amount, suitable irrigation decisions will lead to a situation without water stress. The NWO model under the same conditions shows a total profit of $27,137, which is almost the same as the deterministic results. This result shows that the proposed model is consistent with the NWO model in a deterministic environment. However, both the deterministic model and the NWO model are oversimplified and incorrect by only using the mean of random variables to make decisions. A natural concern arises: what will happen when there is water shortage, and deficit irrigation is therefore needed? For each scenario, assuming sufficient information is available before making decisions (which is a hypothetical setting since we cannot know the weather, yield, and price at sowing season), the wait-and-see decisions could be found. The basic statistics of objective values for these wait-and-see decisions are summarized in Table 2. The average objective value of *WS* solutions is $16,790. These *WS* decisions are not implementable; however, the *WS* solutions are the upper bound of profits under stochastic environments. On the other hand, the objective value of the *EV* problem is based on the assumption of deterministic environments (which is also not realistic). The significant profit drop from *EV* to *WS* indicates that the *EV* problem greatly underestimates the effects of stochastic environments because in the *EV* problem, the first-stage decision is made without considering uncertainty. If *EV* decisions are applied to the stochastic environment, the objective value (profit) of *EEV*^*TS*^ ends up being $12,127, and the performance is not satisfactory, as shown in Table 2.

**Table 2:** Basic statistics for *WS* and *EEV*^*TS*^ objective values (dollars).

	Min.	1st Qu.	Median	Mean	3rd Qu.	Max.
*WS*	0	7,220	18,350	16,790	26,980	38,440
*EEV*^*TS*^	-20,120	1,570	14,940	12,127	22,200	33,850

There is an information gap of $4,663 between the *WS* and the *EEV*^*TS*^ solutions, or the *WS* solution is 38.44% higher than the *EEV*^*TS*^ solution. This gap indicates that applying stochastic programming has potential for better decisions.

### Results based on analysis of two-stage stochastic programming model

**Fig 3:**
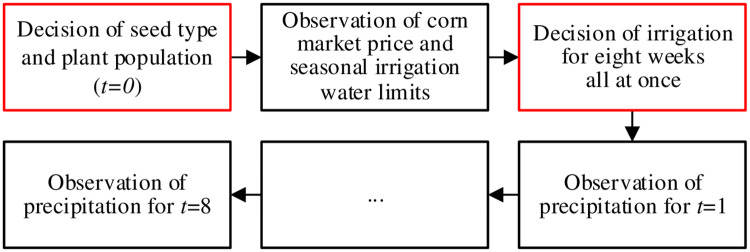
The detailed decision process for two-stage stochastic programming.

Before analyzing the multistage stochastic programming frameworks, the two-stage stochastic programming is first investigated to calculate the *RVSS* and verify the benefits of stochastic programming. Two-stage stochastic programming is a special case of stochastic programming, which has a much shorter decision process. In the two-stage stochastic programming model, the first stage (*t* = 0) still makes pre-season decisions including seed type selection and plant population selection. At the beginning of the second stage (*t* = 1), realizations of corn price and seasonal irrigation water limits become available. The second-stage decisions concern how much irrigation water will be used for the next eight weeks. These second-stage decisions are made all at once at the beginning of the second stage. Note that the precipitation amount for the next eight weeks is not available when making these second-stage decisions, but this precipitation information will be used later in the model to evaluate the objective values. The decision process is summarized in Fig 3.

The constraint in Eq (36) should be changed to the following constraint to reflect the change of decision process:

$$\begin{array}{c} y_{tw}=y_{tw^{\prime }}\;\forall t,\forall w,w^{\prime }\;for\;which\;\xi_{\left[t=2\right]}^{w}=\xi_{\left[t=2\right]}^{w^{\prime }} \end{array}$$ (37)

Note that two-stage stochastic programming is a special case of multistage stochastic programming, in which the decision maker has to make irrigation decisions at an earlier time period. For a maximization problem, the optimal solutions to a multistage problem will have a profit no less than the optimal solution to a two-stage problem because the multistage formulation’s solution can adapt to information as it comes in. In other words, additional stages allow more recourse and will yield to better (at least no worse) decisions.

The objective value of two-stage stochastic programming $\left(v_{f}^{TS}\right)$ is $13,367, which yields a *VSS* of $1,239 and a *RVSS* of 10%. These results show that a 10% profit increase could be achieved by taking corn price and total water limit uncertainties into consideration when making pre-season decisions of seed type selection and plant population selection. Note that the uncertainty of precipitation is ignored in the two-stage decision process. The *EVPI* is $3,423, which also indicates that having additional information could potentially increase profits.

**Fig 4:**
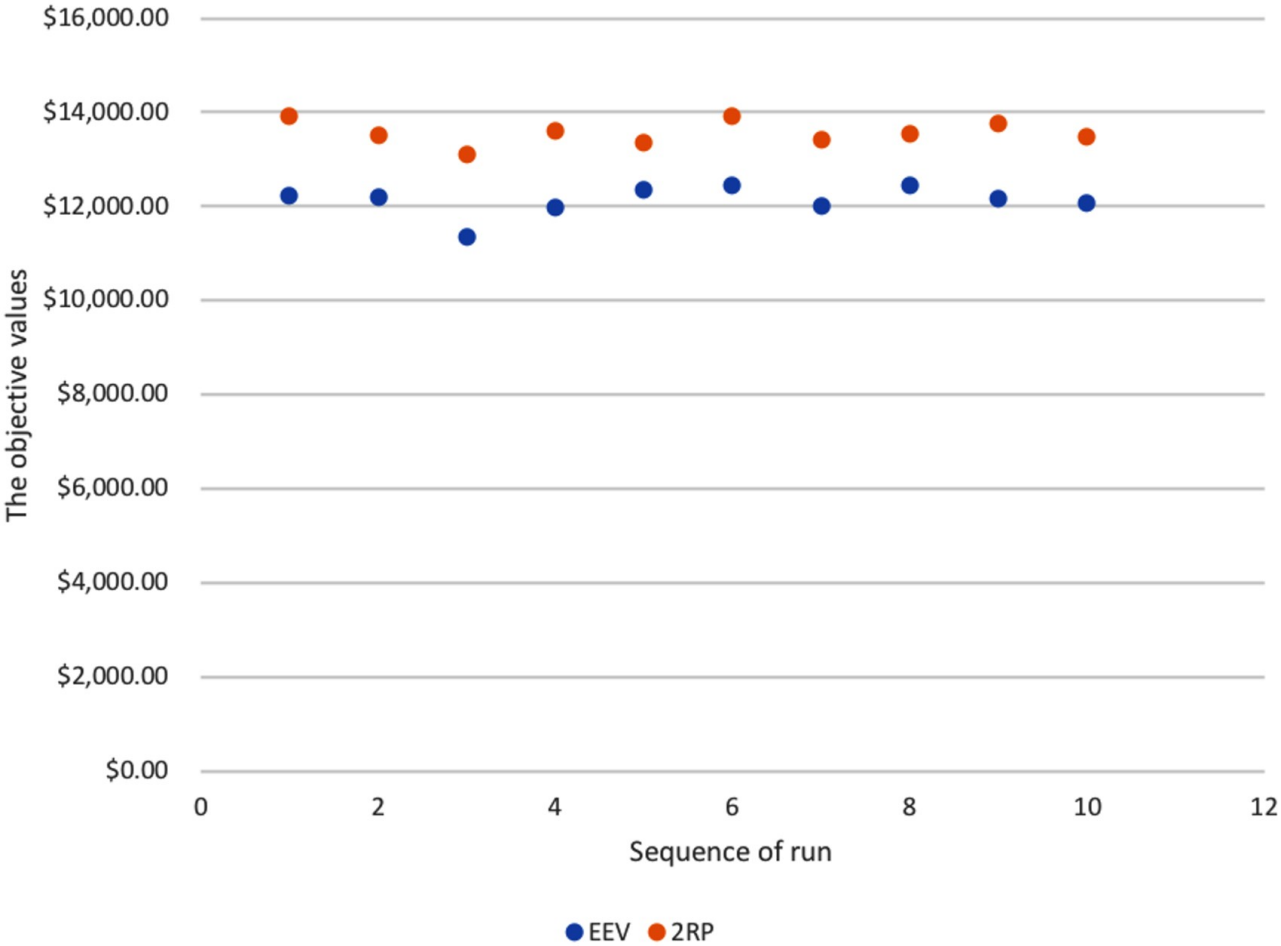
The objective values of two-stage *RP* and *EEV* for ten runs (dollars).

For two-stage stochastic programming, an in-sample stability test is used to test internal consistency of the scenario generation process. The same procedures of scenario generation and model solving are conducted ten times for the stability test. The objective values of the two-stage *RP* and *EEV* are summarized in Fig 4. The $v_{f}^{TS}$ for each time ranges from $13,114 to $13,933. These relatively small ranges indicate that the scenario generation process is in-sample stable.

### Results based on analysis of multistage stochastic programming model

The objective value of the multistage stochastic programming model $\left(v_{f}^{MS}\right)$ is $15,116, which yields a *VMS* of $1,749 and a *RVMS* of 13%. These results could be interpreted as a 13% profit increase will be achieved by taking precipitation uncertainty into consideration and using a multistage decision process when making decisions.

The standard in-sample stability test and out-of-sample stability test are not suitable for multiperiod trees, as nodes beyond the root do not coincide [53]. The weak out-of-sample stability test for multiperiod trees is used to evaluate the stability of the scenario generation process. The procedure of the weak out-of-sample stability test is to build two scenario trees, find the corresponding solutions, and then solve the optimization model on the first scenario tree with the first-stage decisions from the second tree, and vice versa. The model should obtain approximately the same optimal objective values if the method is out-of-sample stable. The two objective values of the multistage stochastic programming model obtained by switching the optimal decision are $15,116 and $15,304, respectively. The result indicates the model has out-of-sample stability.

Table 3 summarizes the profits, decisions, and costs for different models under stochastic environments. Again, the profit for the deterministic model is the *EEV*, meaning that the deterministic model decisions are applied in stochastic environments.

**Table 3:** Comparison among different models (dollars).

Model	Deterministic *EEV*	Two-stage SP	Multistage SP
Total profits	12,127	13,367	15,116
Sales of corn	N/A	97,157	99,456
Production costs	N/A	60,018	60,018
Irrigation costs	N/A	23,797	24,278
Seed selection	High yield	High drought tolerance	High drought tolerance
Plant population	High	High	High
Time for irrigation decision	During sowing	Beginning of irrigation season	Progressively during irrigation season
Uncertainties considered	None	Corn price and water availability	Corn price, water availability, and precipitation

In the stochastic programming results, more conservative first-stage decisions are made such as selecting the high drought-resistant seed. These decisions perform more robustly in stochastic environments. However, all models prefer a high plant population, which indicates that the benefit of increasing yields is more significant than the drawback of seed cost for high plant population. However, a low plant population is still recommended by the seed company at water-limited sites with no irrigation systems. It is worth noting that the changes in seed population (for typical seeding rates) have limited impact on ET. Moreover, regardless of the model assumptions of the water supply constraint and relatively low fixed costs of water abstractions, regulation on water cost is not an effective way to influence farmers’ decisions since water demand for irrigation under deficit irrigation becomes very inelastic. Farmers tend to choose a high plant population and a high irrigation volume.

**Fig 5:**
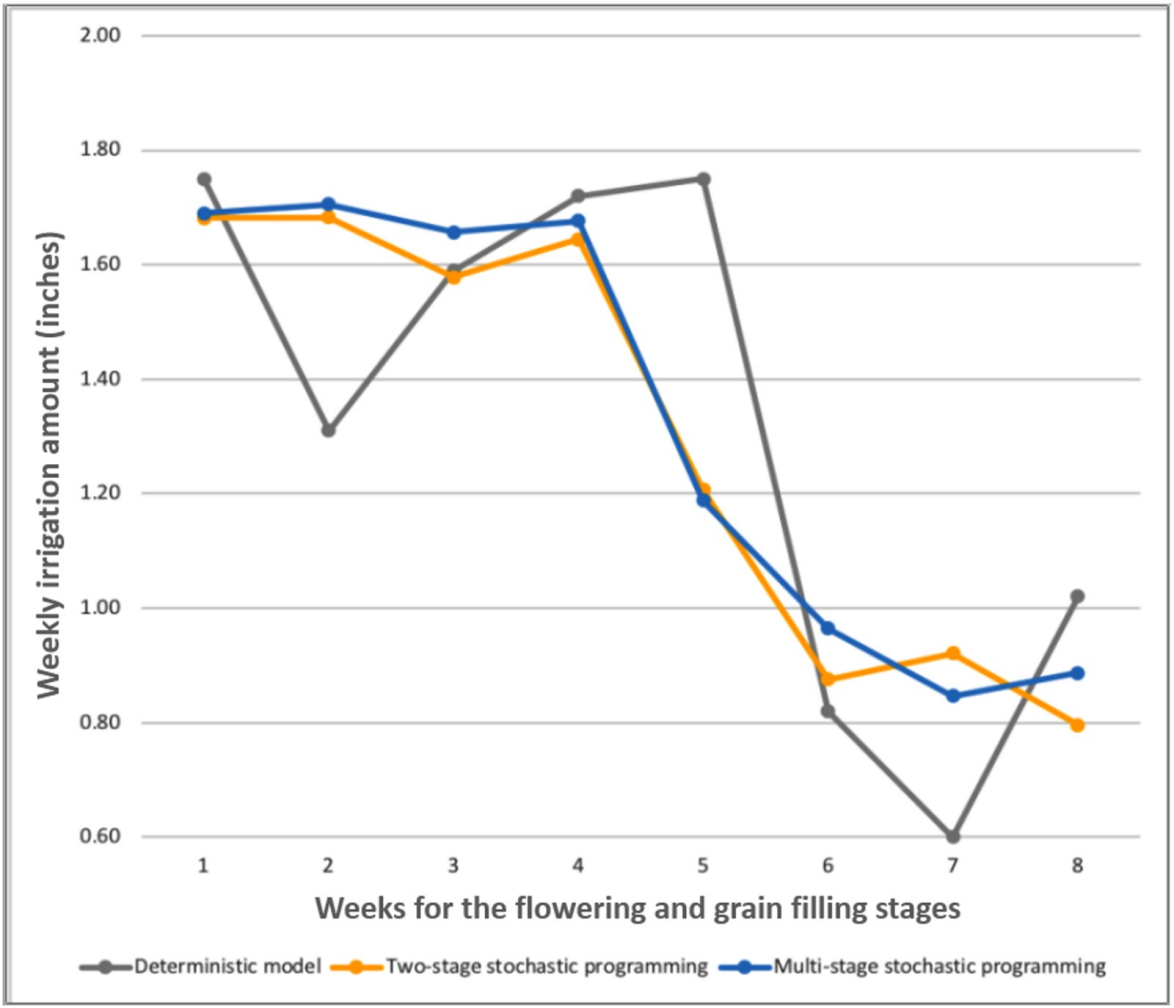
Comparison of weekly irrigation amounts among different models (inches).

Although only the first-stage decisions are implementable and all the later-stage decisions are scenario-based, it is still meaningful to compare the average irrigation amount decisions for each model. Fig 5 summarizes the weekly average irrigation amount from each model. As shown in Fig 5, the irrigation decisions in the deterministic model are very progressive since it assumes the precipitation is deterministic and known. The irrigation decisions for two-stage stochastic programming and multistage stochastic programming models share the same pattern, but the irrigation decisions for the two-stage stochastic programming model are more conservative. This is because little precipitation information is available for the two-stage stochastic programming model when making decisions at the second stage. The multistage stochastic programming model can make recourse irrigation decisions based on the precipitation information.

Note that the information-releasing process is the same for deterministic model, two-stage stochastic programming model, and multistage stochastic programming model. The main difference among these models is the decision-making process. The deterministic model makes all decisions all at once, the two-stage stochastic programming model separates the decision-making process into two stages, and the multistage stochastic programming makes a sequence of decisions according to the stages. The case study results show that by delaying the decision-making process and considering more information (uncertainty), higher profits could be achieved.

## Conclusions

In this study, a multistage stochastic programming model for farmland irrigation management under uncertainty is proposed. The first-stage decisions include pre-season decisions of seed type selection and plant population selection, while the later stages determine irrigation scheduling during the corn flowering and grain-filling stages. The uncertainties under investigation include the corn price, irrigation water limits, and precipitation amount. Their distributions are carefully defined based on a detailed derivation process, and the sample average approximation method is used to generate scenarios.

The case study is based on a farm in Nebraska, U.S.A., which is used to illustrate and validate the optimization model. The numerical results show that a 10% profit increase could be achieved by taking the uncertainties of corn prices and total water limits into consideration, and an additional 13% profit increase could be achieved by also taking precipitation information into consideration. These results indicate that stochastic programming is a promising framework for farm-scale irrigation management under uncertainty and can increase farm profitability significantly.

This study is subject to a number of limitations. First, the numerical results reported in the case study are the best feasible solution in a reasonable computational time. More efficient algorithms and heuristic solutions based on artificial intelligence and meta-heuristic methods such as Genetic Algorithms and Artificial Neural Networks should be investigated. Second, the case study only illustrates the model of a center pivot sprinkler system with almost homogeneous soil features. Other irrigation systems and land profiles could also be investigated. Third, we consider three sources of independent uncertainties, and more dependent stochastic factors can be considered in farm-level irrigation problems. Last but not least, this model focuses on a single-year profit maximization problem. In addition, as suggested by the reviewers, the evaluation of new irrigation system installations in a multi-year horizon considering water allocation policy limitations is another interesting research topic. These topics shall be reserved as future research directions.

## Supporting information

S1 Dataset (XLSX)
